# Naturally Derived Anti-HIV Polysaccharide Peptide (PSP) Triggers a Toll-Like Receptor 4-Dependent Antiviral Immune Response

**DOI:** 10.1155/2018/8741698

**Published:** 2018-07-15

**Authors:** Madeline Rodríguez-Valentín, Sheila López, Mariela Rivera, Eddy Ríos-Olivares, Luis Cubano, Nawal M. Boukli

**Affiliations:** Biomedical Proteomics Facility, Department of Microbiology and Immunology, Universidad Central del Caribe School of Medicine, Bayamón, PR 00960, USA

## Abstract

**Aim:**

Intense interest remains in the identification of compounds to reduce human immunodeficiency virus type 1 (HIV-1) replication. *Coriolus versicolor*'s polysaccharide peptide (PSP) has been demonstrated to possess immunomodulatory properties with the ability to activate an innate immune response through Toll-like receptor 4 (TLR4) showing insignificant toxicity. This study sought to determine the potential use of PSP as an anti-HIV agent and whether its antiviral immune response was TLR4 dependent.

**Materials and Methods:**

HIV-1 p24 and anti-HIV chemokine release was assessed in HIV-positive (HIV+) THP1 cells and validated in HIV+ peripheral blood mononuclear cells (PBMCs), to determine PSP antiviral activity. The involvement of TLR4 activation in PSP anti-HIV activity was evaluated by inhibition.

**Results:**

PSP showed a promising potential as an anti-HIV agent, by downregulating viral replication and promoting the upregulation of specific antiviral chemokines (RANTES, MIP-1*α*/*β*, and SDF-1*α*) known to block HIV-1 coreceptors in THP1 cells and human PBMCs. PSP produced a 61% viral inhibition after PSP treatment in HIV-1-infected THP1 cells. Additionally, PSP upregulated the expression of TLR4 and TLR4 inhibition led to countereffects in chemokine expression and HIV-1 replication.

**Conclusion:**

Taken together, these findings put forward the first evidence that PSP exerts an anti-HIV activity mediated by TLR4 and key antiviral chemokines. Elucidating these new molecular mediators may reveal additional drug targets and open novel therapeutic avenues for HIV-1 infection.

## 1. Introduction

In the early 1980s, the human immunodeficiency virus type 1 (HIV-1) retrovirus was identified as the causative agent of acquired immunodeficiency syndrome (AIDS), one of the most devastating infectious diseases that have emerged in recent history [1, 2]. Viruses have evolved a wide variety of strategies by which they maintain long-term infection of populations. HIV-1's ability to establish reservoirs allows it to maintain a persistent infection, making it difficult to eradicate [3]. Antiretroviral therapies (ART) have been shown to extend the life of patients [4] by preservation of HIV-1-specific CD4+ cells and by lowering the level of HIV-1 viral replication, thereby reducing infectivity and preventing AIDS-related diseases [5]. Although new ART regimens show lower cytotoxicity, these medications are expensive for underprivileged communities [6, 7] and continue to produce side effects [8], leading to adherence and treatment failure [9, 10]. Thus, new nontoxic and inexpensive therapies are needed to reduce HIV-1 infectivity.

*Coriolus versicolor* [11, 12] is an edible fungus of the Basidiomycota family that grows naturally in the bark of trees and is well known for its immune boosting capabilities. One of its most potent alcohol-extracted natural products is polysaccharide peptide (PSP) [13] that has been considered among non-toxic and natural treatments with apotential anti-HIV-1 response [11, 12]. PSP contains the main chain of alpha and beta (1–4) glucans as well as tightly bound polypeptides (15–38% by weight). Due to their branched structures and high molecular weights, beta glucans are powerful immune system builders [14]. PSP, composed of such conserved signature molecules, functions as pathogen-associated molecular patterns (PAMPs).

### 1.1. Polysaccharide Peptide (PSP)

Upon PAMP engagement, the innate immune response is rapidly triggered [15, 16] through the activation of microbial patterns by germline-encoded receptors named pattern recognition receptors (PRRs) [17]. Hence, PSP exerts high potency to initiate and support immune responses with anticancer and potential antiviral capabilities [11, 12, 18]. It has been abundantly demonstrated that HIV-1 patients are in need of immune boosting therapy [19]. In an effort to identify novel anti-HIV-1 agents, we propose to investigate the anti-HIV-1 effects of *Coriolus* PSP and to determine if its antiviral capabilities were Toll-like receptor 4- (TLR4) dependent.

Toll-like receptors (TLRs) are known as PRRs whose specific level of action depends on the infecting pathogen and have central roles in the innate immune system, representing the first line of defense [20–22]. The effect of activating TLRs is typically amplified, giving rise to a signaling cascade that leads to an orchestrated defense mechanism [23]. Previous investigations have demonstrated that PSP stimulates the immune system through the activation of a TLR4 signaling cascade [15], resulting in cytokine and chemokine production [24]. Antiviral chemokines, such as regulated on activation, normal T cell expressed and secreted (RANTES), macrophage inflammatory proteins 1 alpha and beta (MIP-1*α*/*β*), and stromal cell-derived factor 1 alpha (SDF-1*α*), are known to reduce HIV-1 viral replication by targeting viral entry [25, 26]. Past studies have also shown the ability of TLR4 to lower HIV-1 replication [27]; thus, we hypothesized that *Coriolus* PSP possess antiviral capabilities that will lower HIV-1 viral replication through a TLR4-dependent innate immune activation.

The potential capacity of PSP to inhibit HIV-1 reverse transcriptase activity and CD4/Gp120 interaction has been described [12]; however, the before-mentioned study was not performed in a cell-based model and the precise molecular mechanism behind these findings has not yet been fully elucidated. Therefore, further investigations are required to assess the role of PSP in HIV infection. This current study reveals for the first time the promising potential of PSP in THP1 cells and human PBMCs, as an anti-HIV agent, by downregulating viral replication and promoting the upregulation of TLR4, ultimately leading to the production of anti-HIV-1 chemokines (RANTES, MIP-1*α*/*β*, and SDF-1*α*) known to block HIV-1 coreceptors.

## 2. Materials and Methods

### 2.1. Ethics Statement

HIV-1-monoinfected participants were referred by their primary care physicians to the Immunoretrovirus Research Laboratory at Universidad Central del Caribe. All procedures involving human PBMCs were approved by the Universidad del Caribe Institutional Review Board (IRB), and written informed consent was provided by study participants prior to blood collection.

### 2.2. Cell Culture

THP1-Blue-CD14 (THP1) human monocytes (InvivoGen, San Diego, CA) are immortalized human monocytes that carry an NF*κβ*-inducible secreted embryonic alkaline phosphatase (SEAP) reporter gene. Cells were cultured in Roswell Park Memorial Institute (RPMI) 1640 medium (ATCC, Manassas, VA), supplemented with 100 U/ml penicillin and 100 *μ*g/ml streptomycin and 10% heat-inactivated fetal bovine serum (FBS; ATCC, Manassas, VA). Cells were maintained in T-75 culture flasks in a humidified incubator at 37°C and 5% CO_2_.

Human PBMCs were isolated by Histopaque (Sigma-Aldrich, Saint Louis, MO) gradient centrifugation and cultured in RPMI supplemented with 10% FBS and 100 U/ml penicillin and 100 *μ*g/ml streptomycin, as previously described in Boukli et al. [28]. HIV-negative (HIV−) PBMCs were obtained from healthy donors and stimulated with PHA (phytohemagglutinin, 5 *μ*g/ml) (Sigma-Aldrich, Saint Louis, MO) for three days, prior to HIV-1 acute infection. PBMCs were maintained in T-75 cultured flasks in a humidified incubator at 37°C and 5% CO_2_.

### 2.3. *Coriolus* PSP Preparation

*Coriolus* PSP supplement tablets comprise a 28% polysaccharide-to-peptide ratio and a composition of 60.23 mg/g beta-1,3/1,6-glucan (Mushroom Science, Eugene, OR). PSP tablets were diluted (50 mg/ml) in hot endotoxin-free water, and the obtained solution was centrifuged at 2060 ×g for 5 minutes until it was free of insoluble residues. Ethanol 80% (*v*/*v*) was added (1 : 1 PSP-to-ethanol) dropwise to the top of the solution. PSP was collected on top, followed by centrifugation for collection, washed in absolute ethanol, and collected after centrifugation. PSP was dried in a refrigerated vapor trap at −105°C and 0.2 millibar (Thermo Scientific, Waltham, MA) and stored as dried pellets at −20°C. Prior to use, PSP was weighed and dissolved in culture media to achieve a stock concentration of 20 mg/ml.

### 2.4. HIV-1 Infection

THP1 and healthy PBMCs were pelleted and resuspended in culture medium (RPMI 1640, 100 U/ml penicillin, 100 *μ*g/ml streptomycin, 10% FBS) and supplemented with 8 *μ*g/ml hexadimethrine bromide (Sigma-Aldrich, Saint Louis, MO) in order to enhance infection. Viral load analysis of HIV-1 stocks was carried out through HIV-1 p24 RT-PCR using a COBAS Amplicor analyzer (Roche Molecular Diagnostics, Pleasanton, CA). Stocks with viral loads higher than 10^7^ p24 RNA copies/ml were used for acute infection.

*In vitro* and ex vivo HIV-1 acute infection was achieved in THP1 and PBMCs by adding 100 *μ*l of HIV-1 stocks, propagated from dual-tropism HIV-1_ME46_ (NIH AIDS Reagent Program, Bethesda, MD), per 1 million of cells, to achieve an approximate multiplicity of infection of 1.4. HIV-1-infected cells were incubated in a humidified atmosphere at 37°C and 5% CO_2_ for 24 hours. To guarantee equal infection between control and PSP-treated cells, infection was carried out in the same flask and then divided into control and treatment.

### 2.5. Determining the Optimal PSP Concentration

The half maximal effective concentration (EC50) of PSP to be used was determined by measuring the activation of intracellular signaling. This analysis was performed using the QUANTI-Blue assay (InvivoGen, San Diego, CA), which allows the quantification of secreted embryonic alkaline phosphate (SEAP) released into the supernatant of THP1-Blue cells when the transcription factor nuclear factor kappa *β* (NF*κβ*) is activated. Approximately 3 × 10^5^ cells were seeded in a 96-well plate after being acutely infected with HIV-1. Cells were exposed to different concentrations of PSP (50–1000 *μ*g/ml), and viability was measured after being treated twice (at day 0 and day 3) over a six-day period. Following the manufacturer's protocol, 20 *μ*l of supernatant was collected and transferred into a 96-well plate, the QUANTI-Blue solution was added, and the sample was incubated for three hours at 37°C. SEAP quantification was analyzed using SoftMax Pro software (Molecular Devices, Sunnyvale, CA) coupled to a VersaMax Tunable microplate reader (Molecular Devices, Sunnyvale, CA) detecting at 620 nm. EC50 was calculated using the absorbance readings at the highest concentration and comparing it with baseline levels (nonstimulated cells) readings.

### 2.6. Polysaccharide Peptide Cytotoxicity

PSP cytotoxicity in THP1 cells was analyzed by determining cell viability using the percent of alamarBlue (Bio-Rad, Hercules, CA) reduction, as per the manufacturer's protocol. AlamarBlue assay measures the cell capability to convert resazurin into resorufin, providing insight of cell health and viability. This assay was performed in acutely HIV-1-infected and uninfected THP1 cells treated with PSP. Approximately 3 × 10^5^ cells per well were seeded in a 96-well plate. Cells were exposed to different concentrations of PSP (50–1000 *μ*g/ml), and absorbance was measured at zero, three, and six days of treatment using a VersaMax Tunable microplate reader (Molecular Devices, Sunnyvale, CA) detecting at 570 nm. PSP cytotoxicity in both THP1 and human PBMCs was confirmed using tetrazolium MTT 3-(4,5-dimethylthiazol-2-yl)-2,5-diphenyltetrazolium bromide (Sigma-Aldrich, Saint Louis, MO) cell viability assay after a PSP treatment of 200 *μ*g/ml every three days over a 6-day period.

### 2.7. HIV Viral Replication

HIV viral replication was analyzed by measuring HIV p24 core protein in cell culture supernatant by antibody ELISA (detection range of 20–0.312 ng/ml), following the manufacturer's protocol (MyBioSource, San Diego, CA). Concentration of HIV p24 antigen was quantified using SoftMax Pro software (Molecular Devices, Sunnyvale, CA) coupled to a VersaMax Tunable microplate reader (Molecular Devices, Sunnyvale, CA) detecting at 450 nm.

### 2.8. Gene Expression of Antiviral Chemokines

Expression of RANTES, MIP-1*β*, and MIP-1*α* was analyzed using qRT-PCR. RNA was extracted from cell pellets using TRIzol reagent (Life Technologies, Carlsbad, CA) following the manufacturer's protocol. RNA quality and concentration were quantified spectrophotometrically with a NanoDrop 1000 spectrophotometer (Thermo Scientific, Waltham, MA). cDNA was reverse-transcribed from 1 *μ*g of total RNA using the iScript cDNA synthesis kit (Bio-Rad, Hercules, CA). qRT-PCR was performed by means of SYBR Green gene expression assays using 50 nM (Sigma-Aldrich, Saint Louis, MO) primers. Amplification was carried out in a Bio-Rad CFX96 Touch real-time PCR detection system (Bio-Rad, Hercules, CA) using the following program: 10 minutes at 95°C, 50 cycles of 15 seconds at 95°C, and 1 minute at the gene-specific annealing temperature. The gene-specific primers were as follows: MIP-1*β* forward, 5′-CCCTGGCCTTTCCTTTCAGT-3′ and MIP-1*β* reverse, 5′-AGCTTCCTCGCGGTGTAAGA-3′; RANTES forward and 5′-ACAGGTACCATGAAGGTCTC-3′; RANTES reverse, 5′-TCCTAGCTCATCTCCAAAGA-3′ and MIP-1*α* forward, 5′-TTGTGATTGTTTGCTCTGAGAGTTC-3′; and MIP-1*α* reverse and 5′-CGGTCGTCACCAGACACACT-3′. The gene expression level was defined as the threshold cycle number (CT). Mean fold changes in expression of the target genes were calculated using the comparative CT method (RU, 2^−ΔΔCt^). All data were controlled for the quantity of RNA input by GAPDH forward 5′-CTGGGCTACACTGAGCACC-3′ and GAPDH reverse 5′-AAGTGGTCGTTGAGGGCAATG-3′, serving as the endogenous control and for normalization.

### 2.9. Anti-HIV-1 Chemokine Protein Expression

Protein concentrations of antiviral chemokines such as RANTES (detection range of 22.5–5470 pg/ml), MIP-1*β* (detection range of 133.1–32,350 pg/ml), MIP-1*α* (detection range of 90.9–22,090 pg/ml), and SDF-1*α* (detection range of 151–36,720 pg/ml) were analyzed using a Luminex Multiplex Assay, following the manufacturer's protocol (R&D Biosystems, Minneapolis, MN). The concentration of chemokines was determined after incubating 50 *μ*l of samples' supernatant with the chemokine microparticle cocktail and analyzed in a MAGPIX system (EMD Millipore, Billerica, MA).

### 2.10. TLR4 Expression Analysis

Cells were washed with cold FACS buffer (10% FBS, 0.1% sodium azide in PBS) and blocked with negative human serum for 15 minutes. Direct staining was performed with 10 *μ*l of human TLR4-FITC conjugated antibody per million of cells (following the manufacturer's recommendations) (R&D Biosystems, Minneapolis, MN) for 30 minutes and washed with excess FACS buffer to remove the unbound antibody. Expression of TLR4 was analyzed by flow cytometry (BD FACsCanto II) using unstained and isotype controls. Data analysis was performed using FlowJo data analysis software v.10 (Ashland, OR).

### 2.11. Toll-Like Receptor 4 Inhibition

TLR4 was inhibited in HIV-1-treated THP1 cells using the TLR4-specific inhibitor CLI-095 (InvivoGen, San Diego, CA), according to the manufacturer's instructions. Briefly, 2.6 *μ*g/ml CLI-095 was added 5 hours prior to PSP treatment. Cells were incubated at 37°C and 5% CO_2_ during inhibition. After 5 hours, PSP was added at a concentration of 200 *μ*g/ml, following the PSP treatment protocol. TLR4 inhibition was assessed by quantifying cell activation using the QUANTI-Blue assay (InvivoGen, San Diego, CA).

### 2.12. Statistical Analysis

Cytotoxicity assays, flow cytometry, and protein quantification analyses were performed in biological and technical triplicates; similar results were obtained in at least three separate and independent experiments using HIV-1-infected THP1 cells with or without PSP treatment. Human PBMCs were isolated from random HIV-negative and HIV-positive participants. Four separate and independent studies were performed using HIV− and HIV+ PBMCs. Statistical analysis was performed through unpaired *t*-tests, using GraphPad Prism version 7.01 statistical software (GraphPad Software, La Jolla, CA). For experiments of TLR4 expression and inhibition, statistical analysis was performed using one-way analysis of variance (ANOVA). A *p*value ≤ 0.05 was considered statistically significant, and all data is presented as means ± standard error (SEM).

## 3. Results

### 3.1. PSP Is Not Toxic and Activates THP1 Monocytes

**Figure 1:**
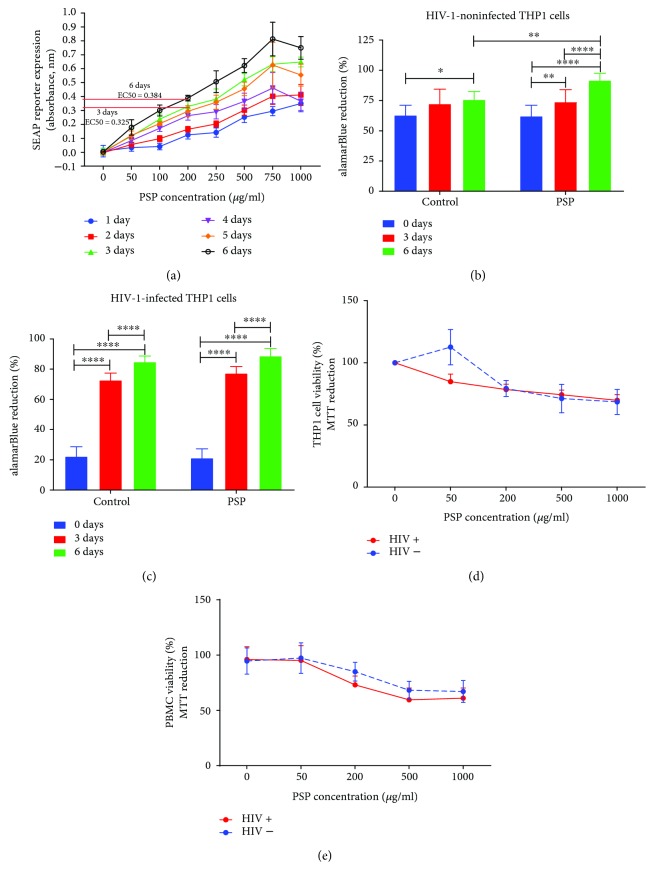
Polysaccharide peptide (PSP) activates human cells without significant cytotoxicity. HIV-1-infected and uninfected THP1 cells and freshly isolated human peripheral blood mononuclear cells (PBMC) were exposed twice (treated at day 0 and day 3) with various PSP concentrations (0–1000 *μ*g/ml) over a six-day period, with 0 *μ*g/ml as negative control. (a) Half maximal effective concentration (EC50) was determined by measuring secreted embryonic alkaline phosphatase (SEAP) activity in the culture supernatant using QUANTI-Blue assay after 6 days of treatment. Reduction of alamarBlue was used to determine the optimal time course between 3 and 6 days of treatments in either (b) HIV-1-negative and (c) HIV-1-positive THP1 cells. Cell viability was confirmed by tetrazolium MTT assay after a six-day period in (d) HIV−/HIV+ THP1 cells and (e) HIV−/HIV+ human PBMCs. Statistical significance was determined using one-way ANOVA, *N* = 3 (^∗^*p* ≤ 0.05, ^∗∗^*p* ≤ 0.01, and ^∗∗∗∗^*p* ≤ 0.0001).

NF*κβ* is a well-recognized transcription factor involved in proinflammatory signaling pathways, including immunomodulatory responses. In order to determine PSP's half maximal effective concentration (EC50), we monitored the NF-*κ*B signal transduction pathway by determining the activity of the secreted embryonic alkaline phosphatase (SEAP). The levels of NF-*κ*B-induced SEAP in the cell culture supernatant are readily assessed with QUANTI-Blue™, a SEAP detection reagent. The assay was performed at different time points of treatment (0–6 days) and different PSP concentrations (0–1000 *μ*g/ml). It demonstrated that PSP treatment at 3 and 6 days were the time points at which PSP provided maximal cell activation. Calculations of EC50 at these two time points revealed that 200 *μ*g/ml was PSP's half maximal effective concentration (Figure 1(a)). PSP toxicity was measured using alamarBlue reduction, a cell-permeable, nontoxic indicator of cell viability and proliferation. The alamarBlue assay was performed at the third and sixth days (3 or 6 days) of treatment in both HIV-1-infected and uninfected THP1 cells. This experiment showed that PSP has no toxic effect in HIV-1 infected or uninfected cells at either 3- or 6-day treatment (Figures 1(b) and 1(c)). However, uninfected cells treated with PSP during a 6-day period showed more viability when compared to control (Figure 1(b)). PSP noncytotoxic effects were confirmed after a six-day treatment using MTT assay in HIV-1 infected and uninfected THP1 cells (Figure 1(d)), resulting in an average 80% of cell viability after 200 *μ*g/ml of PSP (EC50 concentration). Furthermore, PSP-treated THP1 cells maintained a cell viability above 68% in both HIV-1-infected and uninfected cells even in higher concentrations. To obtain a more translational result of PSP nontoxic effects, MTT assay results were validated in human PBMCs, leading to similar results (above 73% viability at 200 *μ*g/ml) (Figure 1(e)). These results suggest that a PSP concentration of 200 *μ*g/ml during a 6-day period is the half maximal effective concentration to achieve a high significant difference in activation of NF*κβ* in THP1 cells without producing major cytotoxic effects (the half maximal inhibitory concentration or IC50 was never reached).

### 3.2. PSP Lowers HIV-1 Replication and Upregulates Antiviral Chemokines

**Figure 2:**
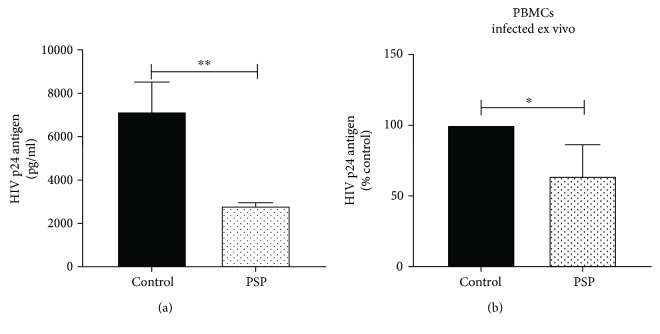
PSP decreases viral replication. HIV-1 viral replication was analyzed after 6 days of PSP treatment (200 *μ*g/ml) by measuring HIV p24 protein in cell supernatants. (a) HIV p24 protein concentration in THP1 cells treated with or without PSP. (b) HIV percentage of inhibition in human PBMCs infected ex vivo and treated with or without PSP from four different healthy subjects. Statistical significance was determined using an unpaired *t*-test, *N* = 3 in THP1 and *N* = 4 in PBMCs (^∗^*p* ≤ 0.05 and ^∗∗^*p* ≤ 0.01).

To determine PSP efficacy as an anti-HIV-1 agent, HIV-1-infected THP1 cells were treated twice with 200 *μ*g/ml PSP over a six-day period. Viral replication in THP1 cell culture supernatant was measured by determining the concentration of the HIV-1 core protein p24. HIV-1 p24 antigen ELISA showed that PSP lowered viral replication in THP1 from an average of 7153 ± 790 pg/ml p24 in control to 2809 ± 85.17 pg/ml in treatments (Figure 2(a)). This result represents an approximately 61% inhibition of viral replication. Given that THP1 cells mimic human monocytes, we wanted to assess PSP capacity to lower viral replication in human peripheral blood mononuclear cells (PBMCs). Four random healthy subjects were recruited following the Institutional Review Board (IRB) protocols. PBMCs were isolated from blood by gradient-density centrifugation, infected with HIV-1 for 24 hours, and treated with 200 *μ*g/ml PSP twice in a 6-day period. PSP lowered viral replication in the four independent studies, ranging from 44.99% to 87.03% viral inhibition (Figure 2(b)), representing an average of 35.99% of viral inhibition.

**Figure 3:**
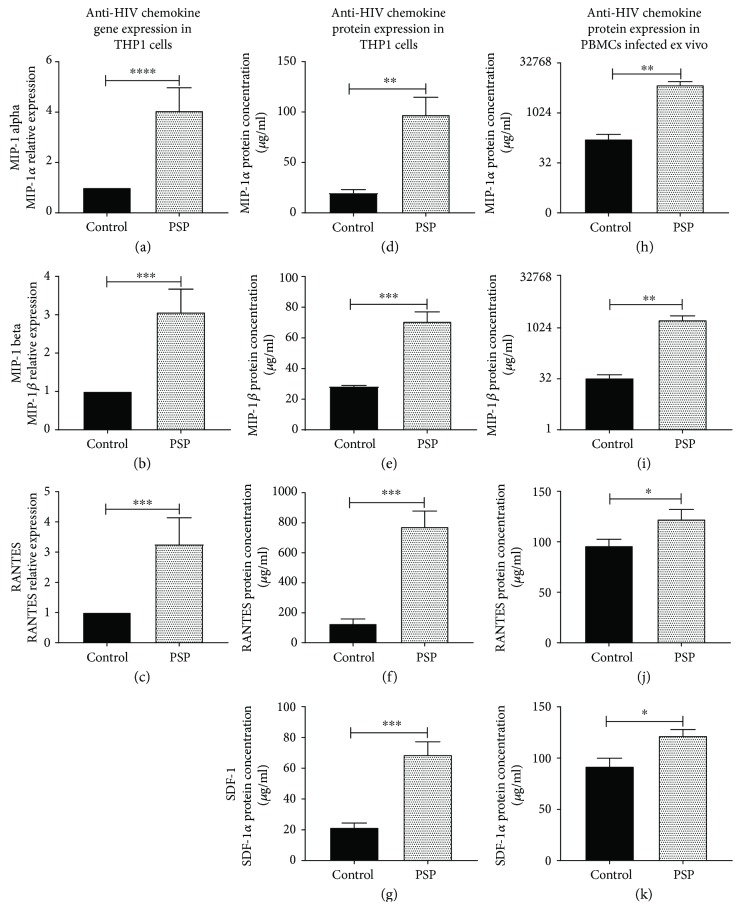
PSP upregulates antiviral chemokines. Anti-HIV chemokine expression was analyzed over a six-day period after being exposed twice with 200 *μ*g/ml PSP. Gene expression of (a) MIP-1*α*, (b) MIP-1*β*, and (c) RANTES was determined in THP1 cells treated with or without PSP using quantitative RT-PCR. Protein concentration of (d) MIP-1*α*, (e) MIP-1*β*, (f) RANTES, and (g) SDF-1*α* were analyzed in THP1 cells' supernatants treated with or without PSP using a Luminex bead array. Chemokine concentrations of (h) MIP-1*α*, (i) MIP-1*β*, (j) RANTES, and (k) SDF-1 were determined in ex vivo infected PBMCs from healthy donors treated with or without PSP by Luminex. Unpaired *t*-test analysis was used to determine statistical significance; *N* = 3 in THP1 and *N* = 4 in healthy human PBMCs (^∗^*p* ≤ 0.05, ^∗∗^*p* ≤ 0.01, ^∗∗∗^*p* ≤ 0.001, and ^∗∗∗∗^*p* ≤ 0.0001).

To further evaluate PSP's immunomodulatory capacity to produce an anti-HIV-1 response, the gene expression of chemokines known to inhibit viral entry such as CCR5's specific chemokines RANTES, MIP-1*α*, and MIP-1*β* were analyzed by quantitative RT-PCR. There was a significant increase of 2.26 ± 0.390 folds in RANTES expression (Figure 3(a)), 3.05 ± 0.409 folds in MIP-1*α* expression (Figure 3(b)), and 2.07 ± 0.301 folds in MIP-1*β* expression (Figure 3(c)) in PSP-treated, HIV-1-infected THP1 cells. The chemokines' anti-HIV bioactivity occurs extracellularly, thus, protein concentration was measured in the cell culture supernatant using a multiplex bead assay with Luminex technology. This experiment showed similar results as the one obtained at mRNA levels, where PSP upregulated the expression of antiviral CCR5-specific chemokines. Cell culture supernatants of PSP-treated THP1 cells showed increased concentrations of MIP-1*α* and MIP-1*β* when compared to control samples (Figures 3(d) and 3(e)). RANTES protein concentration was found to be significantly higher than the other two proteins (Figure 3(f)).

Given that HIV-1 could use either CCR5 or CXCR4 coreceptors in its entry to the cell, we needed to assess if PSP had a direct impact on the expression of a chemokine CXCR4 ligand receptor, SDF-1*α*. Concentration of SDF-1*α* in cell culture supernatant was analyzed by Luminex technology, resulting in 21.45 ± 1.707 *μ*g/ml in control and 68.68 ± 4.918 *μ*g/ml in PSP treated cells (Figure 3(g)).

Additionally, the protein concentration of these antiviral chemokines was analyzed in cell culture supernatants of HIV-1-infected PBMCs obtained from healthy subjects (infected ex vivo). CCR5-specific chemokines were significantly increased in PSP-treated cells. The concentration of MIP-1*α* in PSP-untreated cells (control) was 66.9 ± 45.97 *μ*g/ml and 7120.4 ± 1315 *μ*g/ml in PSP-treated cells, representing a significant increase of MIP-1*α* in PSP-treated cells with a mean difference of 7005 ± 983.2 *μ*g/ml (Figure 3(h)). Similar results were obtained in MIP-1*β*. Control cells had a concentration of 33.72 ± 5.723 *μ*g/ml, while PSP-treated cells had 1741.35 ± 394.8 *μ*g/ml (Figure 3(i)). PSP also upregulated RANTES from 95.99 ± 4.58 *μ*g/ml in control to 122.20 ± 5.633 *μ*g/ml in PSP-treated cells (Figure 3(j)). CXCR4-specific chemokine, SDF-1*α*, was also found to be significantly upregulated in PSP-treated PBMCs infected ex vivo, with a mean difference of 5.98 ± 1.16 *μ*g/ml when compared to control (Figure 3(k)). These results suggest that PSP possess an anti-HIV response and upregulates antiviral chemokines known to inhibit HIV viral entry.

### 3.3. PSP Possesses Potential Translational Capabilities: A Pilot Study Using HIV+ Participants' PBMCs

**Figure 4:**
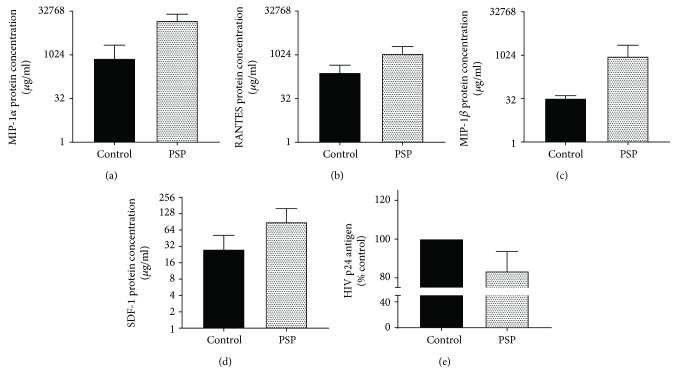
PSP acts as an anti-HIV agent in HIV-infected donors. PBMCs from HIV-1-infected participants were isolated and treated with PSP during a six-day period. Antiviral chemokines (a) MIP-1*α*, (b) RANTES, (c) MIP-1*β*, and (d) SDF-1 concentrations were determined at the end of the treatment in cells' supernatants using Luminex. (e) HIV-1 viral replication was measured by analyzing HIV p24 concentration in PBMCs obtained from HIV-1 participants with and without PSP treatment. PSP untreated cells were used as control. Statistical significance was determined using ratio paired *t*-test analysis, from four different HIV-infected subjects (*N* = 4).

In order to investigate if PSP presents similar results in cells obtained from HIV-1-infected subjects, random HIV-1-positive participants were recruited following IRB's protocols. Four independent studies were carried out by isolating PBMCs, dividing the cells into treatments and controls and incubating with or without PSP (200 *μ*g/ml/6 days). PSP increased antiviral chemokines such as MIP-1*α* (Figure 4(a)) and RANTES (Figure 4(b)) in all four independent studies trending to significance. PSP increased the concentration of MIP-1*β* in three of the four subjects (Figure 4(c)). Furthermore, PSP upregulated SDF-1*α* in three out of four subjects (Figure 4(d)). However, total concentrations of SDF-1*α* were much lower when compared to CCR5-specific chemokines. It is important to emphasize that PSP lowered viral replication in all four independent studies (Figure 4(e)), resulting in an average of 16.54% viral inhibition. These experiments confirm that PSP greatly upregulates chemokines and has potential antiviral capabilities that could be translated into human subjects.

### 3.4. TLR4 Expression Is Upregulated in the Presence of PSP

**Figure 5:**
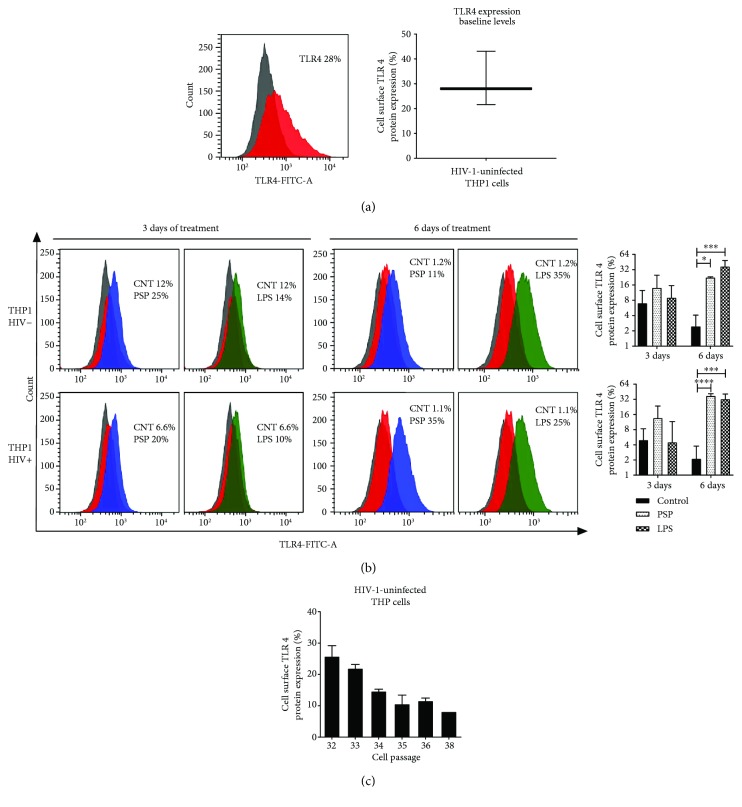
PSP upregulates surface Toll-like receptor 4 (TLR4) expression in human THP1 and peripheral blood mononuclear cells. (a) TLR4 baseline expression (red curves) was determined in HIV− THP1 cells prior to infection and PSP treatment; the IgG isotype was used as negative control (grey curves). Graph represents average of replicates. (b) After 3 and 6 days of 200 *μ*g/ml PSP treatment, surface TLR4 expression was analyzed using direct flow cytometry in either HIV−/HIV+ THP1 cells. Representative images of flow cytometry histograms. Grey curves represent IgG controls, red curves represent control samples (not treated with either PSP or LPS), blue curves represent cells treated with PSP, and green curves represent cells treated with LPS. Bar graphs shows average of replicates, *N* = 3, of both HIV-positive and HIV-negative analysis. (c) TLR4 baseline expression in different cell passages was analyzed using flow cytometry in HIV− THP1 cells; the IgG isotype was used as negative control. LPS was used as a positive control. Statistical analysis was performed using the *t*-test and one-way ANOVA, *N* = 3 (^∗^*p* ≤ 0.05, ^∗∗∗^*p* ≤ 0.001, and ^∗∗∗∗^*p* ≤ 0.0001).

It is well established that the immunomodulatory effects of polysaccharides are related to TLR signaling pathways and that TLR4 plays an important role in the activation of the innate immune response [29, 30] by the production of chemokines [31, 32]. To analyze whether TLR4 is involved in PSP antiviral molecular mechanism, TLR4 expression was measured by flow cytometry at baseline levels in uninfected and untreated THP1 cells, resulting in an average of 32.1% TLR4 surface expression (Figure 5(a)). Results obtained in control at 3 and 6 days after PSP treatment, in HIV-infected and uninfected cells, were questionably low. Additional experiments showed that TLR4 surface expression was lost over time after several cells' passaging in the THP1-Blue cells (Figure 5(c)). However, PSP was able to reestablish baseline TLR4 levels in THP1 cells at the end of the six-day treatment when compared to control (Figure 5(b)).

**Figure 6:**
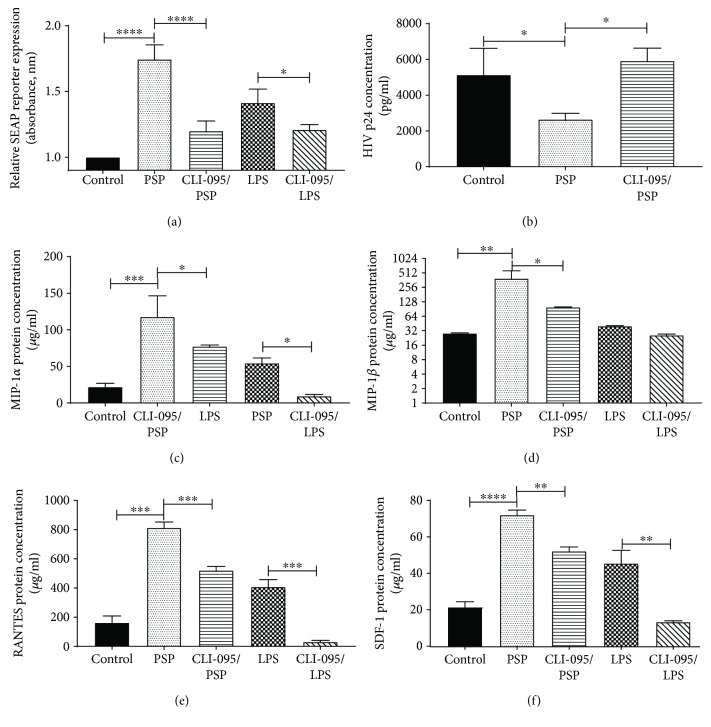
TLR4 is involved in PSP antiviral response. (a) TLR4 activation (through NF*κβ* activation) was determined by measuring the relative expression of the secreted embryonic alkaline phosphatase (SEAP) reporter. Samples were treated with TLR4 inhibitor (CLI-095) and exposed to either of the agonists (200 *μ*g/ml PSP or 10 ng/ml LPS); control cells were untreated HIV-infected THP1. (b) HIV viral replication was measured by analyzing HIV-1 p24 concentration in THP1 cells that were TLR4-inhibited or not with CLI-095 and treated with PSP (as compared to non-TLR4-inhibited/non-PSP-treated cells). Protein concentrations of (c) MIP-1*α*, (d) MIP-1*β*, (e) RANTES, and (f) SDF-1 were determined in THP1 cell supernatants treated with or without PSP using Luminex bead array. LPS and LPS-CLI-095 were used as positive control of TLR4 inhibition. Statistical significance was determined using one-way ANOVA, *N* = 3 (^∗^*p* ≤ 0.05, ^∗∗^*p* ≤ 0.01, ^∗∗∗^*p* ≤ 0.001, and ^∗∗∗∗^*p* ≤ 0.0001).

To achieve a better understanding of PSP action on TLR4, cells were exposed to CLI-095 (also known as TAK 242), a TLR4-specific inhibitor. NF*κβ* activation was analyzed by measuring the SEAP reporter released by THP1-Blue cells (Figure 6(a)). Cells exposed to the TLR4 inhibitor showed decreased activation when compared to cells treated with PSP and without CLI-095. LPS in the presence or absence of CLI-095 was used as positive control of TLR4 activation and inhibition. These findings indicate that PSP stimulates THP1 cells by TLR4.

To confirm TLR4 actions on PSP antiretroviral activity, viral load was measured in cell culture supernatant by HIV-1 p24 antigen ELISA after TLR4 inhibition. PSP lowered viral replication significantly when compared to control, resulting in a 48.5% of viral inhibition. However, TLR4-inhibited cells treated with PSP maintained HIV-1 p24 levels similarly to control cells, suggesting that TLR4 is an important receptor in PSP antiretroviral activity (Figure 6(b)). To further confirm whether TLR4 plays a role in PSP antiviral activity, antiviral chemokines' concentration levels were assessed after TLR4 inhibition. LPS in the presence and absence of CLI-095 was used as positive control. Inhibition of TLR4 resulted in decreased expression of all anti-HIV chemokines. MIP-1*α* protein concentration was reduced in PSP-TLR4-inhibited cells (Figure 6(c)). Similar results were obtained after analyzing MIP-1*β* (Figure 6(d)), RANTES (Figure 6(e)), and SDF-1*α* (Figure 6(f)) in PSP-TLR4-inhibited cells. Ultimately, these results suggest that PSP stimulated THP1 cells via TLR4 and that the engagement of this receptor induced an antiviral response.

## 4. Discussion

Despite years of research and initiatives to develop new antiretroviral medications, HIV-1 is still a menace to humanity [33]. The antiviral available drugs have proven to prolong life expectancy by reducing viral replication [4, 5, 10] but come with serious side effects resulting in a varying degree of toxicity and symptomatology from mild discomfort and inconvenience to permanent damage and disability [8, 34–36]. HIV-1 pathogenesis thrives on the failure of the immune system to control viral replication [37, 38]. Since the innate immune system constitutes the first line of response against invading pathogens, it is of great importance to identify natural immunomodulators specially associated with the innate immune system to be used in combination with classical therapies against HIV-1 replication.

The present study sought to determine the antiretroviral activity of the natural and accessible supplement, *Coriolus* PSP, obtained from the mushroom *Coriolus versicolor*. The mushroom extract-derived, PSP, has demonstrated to possess immune-boosting capabilities and proven to successfully modulate anticancer immunity [11, 15, 18, 39]. Furthermore, it has been used in clinical trials as a cancer medication and has been demonstrated to be effective without having adverse reactions [40]. Tsang et al. [40] showed that PSP increases white blood cell count without the detrimental side effects seen in antiretroviral therapies (ART), confirming the results of other investigators that have demonstrated that PSP increased CD4+ cells and CD4+/CD8+ ratio in esophageal and gastric cancer [41]. This specific quality of PSP in cancer treatment is a powerful characteristic pertaining to our investigation, given that one outcome of HIV-1 pathogenesis is CD4+ depletion and diminished CD4+/CD8+ ratio in HIV-infected patients, suggesting immune dysfunction [42, 43]. Cytotoxicity assays performed during our investigation corroborated Tsang et al.'s results, as 200 *μ*g/ml PSP did not cause a significant toxicity in human THP1 and peripheral mononuclear cells. Moreover, *Coriolus* PSP concentrations higher than 200 *μ*g/ml did not cause toxicity enough to reach the half-maximal inhibitory concentration (IC50), normally used to determine *in vitro* optimal treatment concentrations. The average retroviral inhibition achieved by two doses of PSP in acute HIV-1-infected THP1 human monocytes was 61%, with no significant adverse effects in cytotoxicity. Previous experiments by Collins and Ng [12] reported PSP's ability to inhibit the interaction of HIV-1gp120 and immobilized CD4 receptor, suggesting that PSP blocks this interaction by binding to either protein. Similarly, Fu et al. [44] investigated compounds from rose (*Rosa rugosa*) flowers, including P1, a polysaccharide-peptide complex. This peptide ability to inhibit human immunodeficiency virus type-1 reverse transcriptase (HIV-1 RT) *in vitro* was performed by a cell-free experimental approach, while our study is the first to investigate PSP's immunomodulatory effect against HIV via TLR4 activation by cell-based experimentation. Furthermore, the present study's data demonstrated that PSP induces the production of chemokines, such as MIP-1*α*/*β*, RANTES, and SDF-1*α*, widely known as blockers of HIV-1 coreceptors [26, 45, 46].

HIV-1 coreceptors CCR5 and CXCR4 and their ligands constitute a chemokine/receptor axis that has attracted great interest, partly since both CCR5 and CXCR4 are targets for HIV binding and entry into cells [47, 48]. A significant increase in the expression of such chemokines upon PSP treatment on human HIV-1-infected monocytes and peripheral mononuclear cells creates an environment with high antiviral activity, reflecting the resistance of the immune system against viral infection. Even though HIV-1 has the capacity to adapt and switch its coreceptor tropism [49], PSP has proven to not only promote the production of CCR5-specific antagonists but also stimulate SDF-1*α*, a CXCR4-specific antagonist. Collectively, these findings suggest that PSP inhibits HIVgp120/CD4 receptor, by engaging with the overproduction of HIV coreceptor antagonists, indirectly influencing HIV-1 entry. Ex vivo experiments were used to validate the *in vitro* HIV-1-infected model. The preliminary study performed on PBMCs obtained from four HIV-1-infected participants points to a potential PSP-induced antiviral activity by specifically lowering HIV-1 viral replication and upregulating antiviral proteins. This translational pilot study, while small, indicates promise that PSP treatment leads to an antiviral activity against HIV. Beside the *in vitro* validation of PSP anti-HIV activity, we believe that the ex vivo experiments represent a good starting point to prepare for future studies with a greater number of subjects. These results suggest a trending significance due to the obtained low *p* values (near 0.10–0.05) in such small number of individuals with potential compromised immune system.

The present study not only revealed the potential use of *Coriolus* PSP as an anti-HIV agent but also highlights the novel underlying mechanism by which PSP induces a TLR4-dependent antiretroviral signaling response. Consistent with our results, Wang et al. [15] study demonstrated PSP's ability to trigger TLR4, and Swaminathan et al. [27] have shown that TLR4 was one of the two most potent Toll-like receptors to counteract HIV infection in human macrophages, strongly suggesting that TLR4 implication against HIV-1 infection is of great importance. Additionally, new approaches for HIV vaccines have opted to use TLR4 agonists as adjuvants to promote immune responses due to its potent immunomodulatory activity in innate and adaptive immunity [50–52]. Our inhibitory studies using CLI-095, a cyclohexane derivative that inhibits TLR4 by binding to cysteine 747 of its intracellular domain without affecting the extracellular binding domain [53–56], demonstrate for the first time that TLR4 is a key player in PSP's anti-HIV response. It is important to note that NF*κβ* activation and the expressions of antiviral chemokines (RANTES, MIP-1*α*, MIP-1*β*, and SDF-1*α*) in TLR4-inhibited cells were significantly decreased when compared to PSP-treated cells without inhibition. While other studies reported that HIV-1 infection hijacks NF*κβ* to subsequently stimulate its pathogenesis [57], our work has provided novel insights suggesting an important role of PSP's antiviral response triggered by the TLR4-NF*κβ* signaling axis. Importantly, HIV-1 viral load was increased to normal or higher levels when TLR4 signaling activation was inhibited, even when THP1-cultured cells were exposed to equal concentrations of PSP. However, additional analysis of long-term exposure of HIV-infected cells to PSP are needed to determine its capabilities as long-term treatment and to investigate NF*κβ*'s roles in PSP's antiviral response.

**Figure 7:**
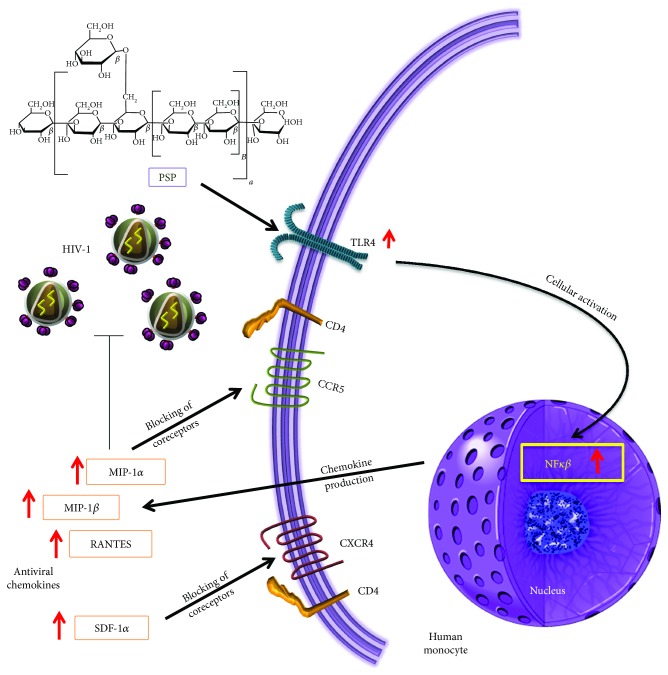
PSP activates TLR4 anti-HIV chemokine production. This model summarizes the findings of this study, suggesting that PSP triggers TLR4 leading to the activation of the transcription factor NF*κβ*. PSP's immune activation induces the overproduction of antiviral chemokines (RANTES, MIP-1*β*, MIP-1*α*, and SDF-1), which are well known to block HIV-1 coreceptors CCR5 and CXCR4 and subsequently downregulate HIV-1 viral replication.

Current results, therefore, confirm our hypothesis and reveal PSP potential use as an anti-HIV-1 agent, mediated by TLR4 early immune response. These congruent data led us to propose a mechanistic model (Figure 7) highlighting that upon PSP exposure, the cell triggers a signaling cascade involving TLR4-mediated NF-*κ*B signaling and its subsequent production of antiviral chemokines (MIP-1*α*, MIP-1*β*, RANTES, and SDF-1*α*). The antiviral environment created by this signaling cascade would allow the immune system to repress HIV-1 replication by blocking viral entry through the CCR5 and CXCR4 coreceptors. The overall implications of these findings suggest that PSP induces an anti-HIV-1 response mediated by TLR4 activation and antiviral chemokine response.

## 5. Conclusion

PSP possesses antiviral activity that contributes to HIV-1 inhibition, through the upregulation of chemokines that are well described as anti-HIV entry proteins. A translational pilot study consisting of PBMCs obtained from HIV-1-infected and uninfected donors showed a clear trend of the anti-HIV role of PSP, demonstrating that PSP's capabilities could be translated to *in vivo* experimentations. Such studies with a larger population from healthy and HIV-infected subjects should be conducted in different stages of HIV infection, as assessed using viral load, and taking the occurrence of antiretrovirals into consideration. Altogether, data obtained from this study support that PSP can provide a natural alternative to inhibit HIV-1 replication. Moreover, these novel findings put forward the first evidence that TLR4 contributes to PSP effects on anti-HIV chemokine release and viral downregulation. Further research will be performed to uncover other possible mechanisms of action, including analyzing the activating capacity of other TLRs.

### 5.1. Future Directions

The implications of assessing PSP's capacity to fight HIV-1 as a long-term treatment and to investigate its potential as an adjuvant medication against HIV-1 infection are potentially profound. Although part of this study was performed in a smaller sample of human PBMCs from healthy (infected ex vivo) and HIV-1-infected donors, a clear trend of anti-HIV's role of PSP was observed. Utilizing these results, such studies will be carried out with a larger population of healthy and HIV-infected subjects.

## Data Availability

The data used to support the findings of this study are included within the article.

## Additional Points

(i) Two exposures of PSP (200 *μ*g/ml) in THP1 cells during a 6-day period is the EC50 to achieve a high significance level in NF*κβ* activation without producing a significant cytotoxic effect. (ii) PSP inhibited 61% of HIV-1 viral replication in THP1 cells after only two exposures of 200 *μ*g/ml during a 6-day period. (iii) PSP significantly induced the upregulation of anti-HIV entry chemokines: RANTES, MIP-1*α*, MIP-1*β*, and SDF-1*α*. (iv) Experiments performed in human PBMCs obtained from HIV-1-infected and uninfected donors revealed 16% and 36% of viral inhibition, respectively. (v) Expression of anti-HIV chemokines was increased in human PBMC samples exposed to PSP. (vi) PSP was able to increase TLR4 surface expression in THP1 cells. (vii) Inhibition of TLR4 induced countereffects of PSP actions in chemokine expressions and HIV-1 viral replication. (viii) PSP demonstrated to induce an immune response against HIV-1, through TLR4 and anti-HIV chemokine release.
